# A Comparative Chemical Study of Calcium Silicate-Containing and Epoxy Resin-Based Root Canal Sealers

**DOI:** 10.1155/2016/9808432

**Published:** 2016-12-20

**Authors:** Przemysław Reszka, Alicja Nowicka, Mariusz Lipski, Włodzimierz Dura, Agnieszka Droździk, Krzysztof Woźniak

**Affiliations:** ^1^Dental Practice, ul. Arki Bożka 32, 75-365 Koszalin, Poland; ^2^Department of Conservative Dentistry and Endodontics, Pomeranian Medical University of Szczecin, Al. Powstańców Wlkp. 72, 70-111 Szczecin, Poland; ^3^Department of Preclinical Conservative Dentistry and Preclinical Endodontics, Pomeranian Medical University of Szczecin, Al. Powstańców Wlkp. 72, 70-111 Szczecin, Poland; ^4^Department of General Dentistry, Pomeranian Medical University of Szczecin, Al. Powstańców Wlkp. 72, 70-111 Szczecin, Poland; ^5^Department of Orthodontics, Pomeranian Medical University of Szczecin, Al. Powstańców Wlkp. 72, 70-111 Szczecin, Poland

## Abstract

*Objective.* The present study assessed the chemical elements in two novel calcium silicate-containing root canal sealers, BioRoot RCS and Well-Root ST, compared to a calcium silicate-containing root canal sealer that has been on the market for several years, MTA Fillapex, and epoxy resin-based sealer AHPlus.* Material and Methods.* The sealers were mixed and manipulated according to the manufacturers' instructions. Twelve cylindrical molds (inner diameter 4 mm; height 3 mm) were placed on a glass petri dish and packed with the materials. The dish was transferred to an incubator. After 72 h the molds were examined by scanning electron microscopy and energy dispersive X-ray microanalysis.* Results.* BioRoot RCS and Well-Root ST had high peaks of calcium, zirconium, oxygen, carbon, silicon, and chlorine. Well-Root ST also had sodium, magnesium, aluminum, and titanium peaks. MTA Fillapex and AHPlus had carbon, oxygen, calcium, titanium, and bismuth peaks. A silicon peak was also observed for MTA Fillapex, and zirconium and tungsten peaks for AHPlus.* Conclusion.* BioRoot RSC had the highest degree of purity. The clinical implication of metals contained in the other sealers needs to be investigated.

## 1. Introduction

Filling of the root canal involves the use of core material, such as gutta-percha, in combination with root canal sealer to provide an adequate seal. The primary role of the sealer is to obliterate irregularities between the root canal wall and the core material [1–4]. Root canal sealers, even if they will not be extruded beyond the apical foramen, are in direct contact with periodontal ligament and bone over extended periods of time and may release toxic elements, irritating these tissues and influencing the final outcome of the root canal. Therefore, a study of the chemical characteristics of root canal sealers is desirable [5].

Root canal sealers can be grouped based on their prime constituent or chemical structure, such as zinc oxide-eugenol, calcium hydroxide, silicone, glass ionomer, and epoxy or methacrylate resins. Recently, a new type of sealer containing mineral trioxide aggregate and calcium silicate has been developed. An advantage with these novel sealers is their potential bioactive properties. Similar to other silicate-containing materials, Ca(OH)_2_ is produced upon reaction with water, leading to a high alkaline pH that activates and stimulates the expression of alkaline phosphatase, favoring the formation of mineralized tissue and having an antimicrobial effect. In addition, the alkaline pH could neutralize the lactic acid from osteoclasts and prevent dissolution of the mineralized components of teeth [6–9].

One of the first mineral trioxide aggregate-containing root canal sealers introduced on the market was MTA Fillapex (Angelus, Londrina, Brazil). Because it has been available for 5 years, it is the most studied MTA-containing root canal sealer. MTA Fillapex is a paste-catalyst material. Paste A is composed of salicylate resin (methyl salicylate, butylene glycol, and colophony), bismuth oxide, and silica. Paste B includes silicon dioxide, titanium dioxide, and base resin (pentaerythritol, rosinate, and toluene sulphonamide), and 13.2% set MTA particles as filler. The working time is 23 min, with a complete set time of approximately 2 h. Several properties of this root canal sealer, such as flow and viscosity [10, 11], dimensional change [11], material porosity [12, 13], sealing ability [11], radiopacity, electrical conductivity [14], antibacterial effect [15], biocompatibility [16–20], cytotoxicity [19, 21–23], and genotoxicity [24, 25], have been investigated.

Another recently introduced sealer based on tricalcium silicate is Well-Root ST (Vericom, Gangwon-Do, Korea). This sealer is a premixed, ready-to-use, injectable bioceramic cement paste developed for permanent obturation of the root canal. The composition of Well-Root as described by the manufacturer includes zirconium oxide, calcium silicate, filler, and thickening agents [26]. The material is hydrophilic and uses moisture in dentinal tubules to initiate and complete its setting reactions. The setting time is 25 min, but in root canals the setting time can be more than 2.5 h. According to the manufacturer, the Well-Root ST should be used in conjunction with gutta-percha points. No information on the chemical composition and physical properties of this root canal sealer is available in the current scientific literature.

A new tricalcium silicate-based root canal sealer was introduced recently. BioRoot RCS (Septodont, Saint Maur-des-Fosses, France) consists of a powder and a liquid. The powder is composed of tricalcium silicate, zirconium dioxide, and povidone, and the liquid is composed of water, calcium chloride, and polycarboxylate. The BioRoot RCS has a minimum working time of 10 min and a maximum setting time of 4 h. This silicate-based root canal sealer has less toxic effects on human periodontal ligament cells than zinc oxide-eugenol sealer and induces a higher secretion of angiogenic and osteogenic growth factors than ZOE [27]. BioRoot RCS compared to contemporary root canal sealers (AHPlus, Acroseal, EndoRez, RealSeal SE, Hybrid Root SEAL, RootSP, and MTA Fillapex) has the lower cytotoxicity and genotoxicity [24]. The sealing properties of BioRoot RCS combined with gutta-percha are comparable to those of AHPlus, but microCT has revealed a higher void volume for BioRoot RCS than resin-based sealer, possibly due to the shorter working time and less flow than AHPlus [28].

AHPlus (Dentsply, DeTrey, Konstanz, Germany) is an extensively studied epoxy resin-based sealer and considered a gold standard endodontic sealer. The material is composed of epoxy resin, calcium tungstate, aerosil, iron oxide, adamantane amine, N,N-dibenzyl-5-oxanonane, TCD-diamine, calcium tungstate, and zirconium oxide.

Because of the good biocompatibility of bioceramic cements, calcium silicate-based root canal sealers are increasingly used for permanent root canal filling (BioRoot RCS, Septodont, Saint Maur-des-Fosses, France; Endo-CPM sealer, EGEO, SRL, Buenos Aires, Argentina; Endo Sequence BC Sealer, Brasseler Savannah, GA; iRoot, Innovative Bioceramix Inc., Vancouver, Canada, MTA Fillapex, Angelus, Londrina, Brazil; ProRoot ES Endo Root Canal Sealer, Dentsply Tulsa, Johnson City, TN; Tech Biosealer, Isasan, Rovello Porro, Italy; Well-Root ST, Vericom Co., LTD, Gangwon-Do, Korea). However, several studies have shown that some of these materials may cause cellular degeneration and delayed wound healing of periapical tissues [29, 30]. The cytotoxic effect of these endodontic sealers may be caused by heavy metals released from the set materials [11, 31].

Many studies have evaluated the chemical elements and heavy metals in MTA Fillapex and AHPlus [14, 29, 30] but, to the best of our knowledge, no studies have chemically analyzed the two new calcium silicate-containing root canal sealers, BioRoot RCS and Well-Root ST. The aim of the present study was to determine the chemical elements in these novel calcium silicate-containing root canal sealers. The results were compared to a calcium silicate-containing root canal sealer that has been on the market for several years MTA Fillapex and epoxy resin-based sealer AHPlus.

## 2. Material and Methods 

The following root canal sealers were used in this study:

1. BioRoot RCS (Septodont, Saint Maur-des-Fosses, France)
2. Well-Root ST (Vericom, Gangwon-Do, Korea)
3. MTA Fillapex (Angelus, Londrina, Brazil)
4. AHPlus (Dentsply, DeTrey, Konstanz, Germany).

### 2.1. Sample Preparation

All sealers were mixed and manipulated according to the manufacturers' instructions. For scanning electron microscopy (SEM), twenty cylindrical molds with an inner diameter of 4 mm and height of 3 mm were placed on a glass petri dish and packed with the materials. Five homogeneous specimens were made for each studied material. The dish was then covered with wet gauze and transferred to an incubator (37°C, 95% relative humidity). After 72 h the specimens were ground by progressively finer diamond discs and pastes using a polishing machine.

### 2.2. Scanning Electron Microscopy and Energy Dispersive Microanalysis

The materials were examined using the following methods: SEM with a FE-SEM Hitachi SU-70 microscope, energy dispersive spectroscopy (EDS) X-ray microanalysis using NORAN™ System 7 UltraDry X-ray detector (Thermo Fisher Scientific). All analyses were performed at an accelerating voltage of 25 kV and electron beam current of approximately 3 nA. The “PROZA” correction method was applied for quantitative EDS analyses. The estimated uncertainty for EDS measurements was 0.1 wt.%. Samples were coated with a very thin coating of gold-palladium alloy for electric conductivity. The metals used to sputter coat the specimens were excluded from the percentage found. Backscattered electron images in compositional contrast were acquired.

## 3. Results

**Figure 1:**
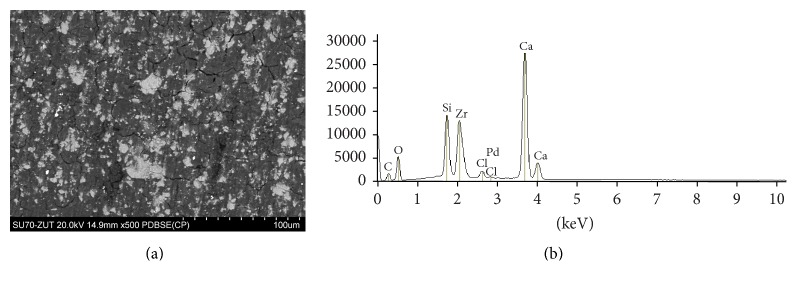
BioRoot RCS: backscatter scanning electron micrographs at 500x magnification (a); EDS X-ray microanalysis (b).

**Figure 2 fig2:**
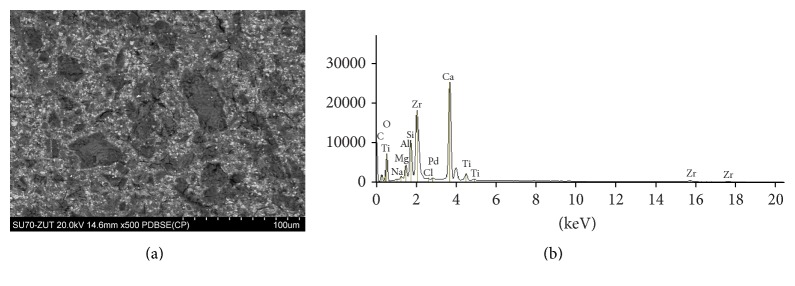
Well-Root ST: backscatter scanning electron micrographs at 500x magnification (a); EDS X-ray microanalysis (b).

**Figure 3 fig3:**
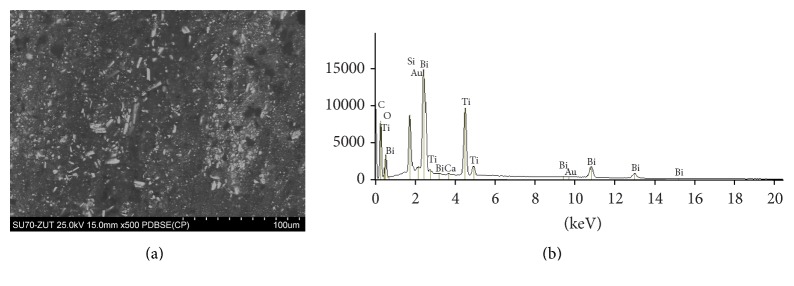
MTA Fillapex: backscatter scanning electron micrographs at 500x magnification (a); EDS X-ray microanalysis (b).

**Figure 4:**
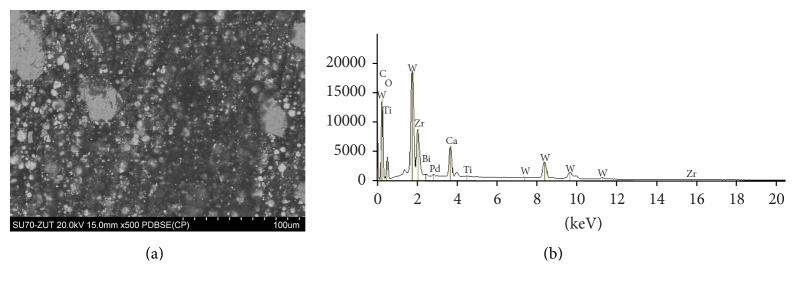
AHPlus: backscatter scanning electron micrographs at 500x magnification (a); EDS X-ray microanalysis (b).

The SEM images and EDS profiles for the randomly selected areas of equal sizes of the studied root canal sealers are shown in Figures 1 [Fig fig2] [Fig fig3]–4. Quantitative results of elements according to microanalysis are described in Table 1. EDS microanalysis of BioRoot RCS and Well-Root ST revealed high peaks for calcium, zirconium, oxygen, silicon, carbon, and chlorine. For Well-Root ST, peaks were also present for sodium, magnesium, aluminum, and titanium. MTA Fillapex and AHPlus had peaks for carbon, oxygen, calcium, titanium, and bismuth. For MTA Fillapex, a peak was also present for silicon, and for AHPlus peaks were present for zirconium and tungsten.

**Figure 5:**
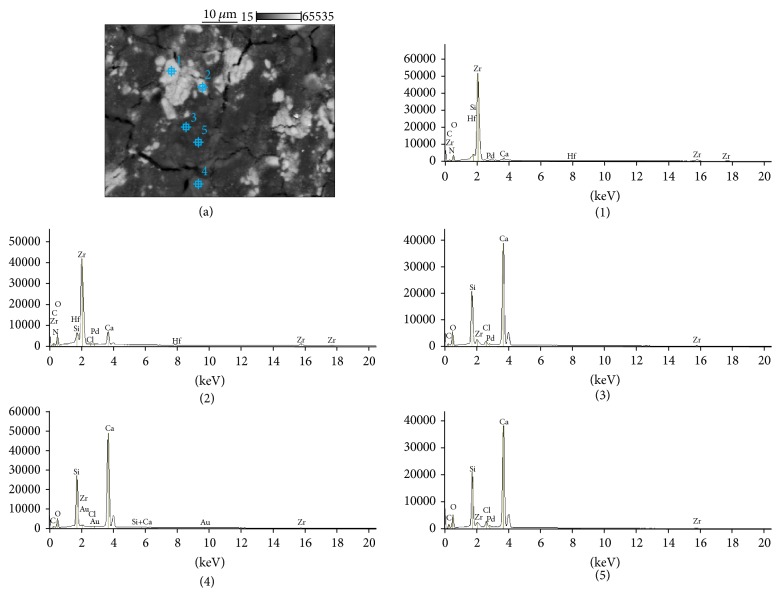
BioRoot RCS: backscatter scanning electron micrographs at 1000x magnification (a); EDS X-ray microanalysis of particles and cementation phase (1, 2, 3, 4, and 5).

**Figure 6:**
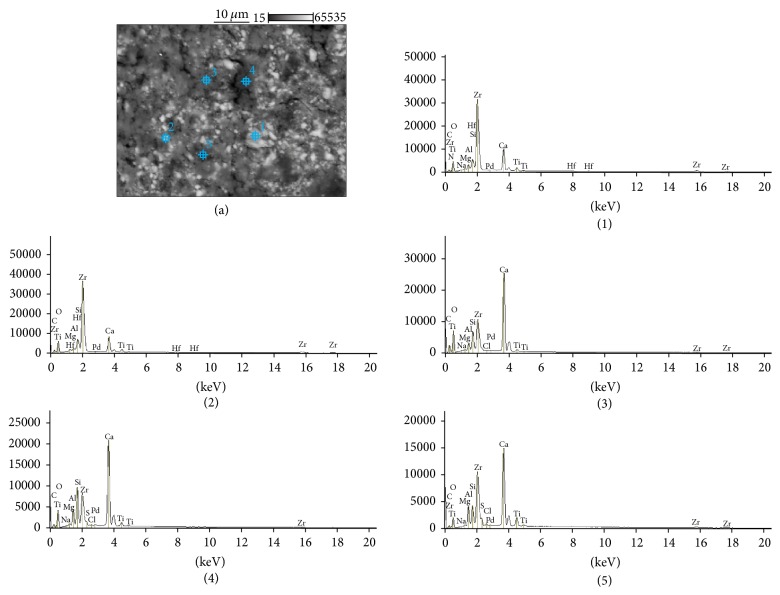
Well-Root ST: backscatter scanning electron micrographs at 1000x magnification (a); EDS X-ray microanalysis of particles and cementation phase (1, 2, 3, 4, and 5).

**Figure 7:**
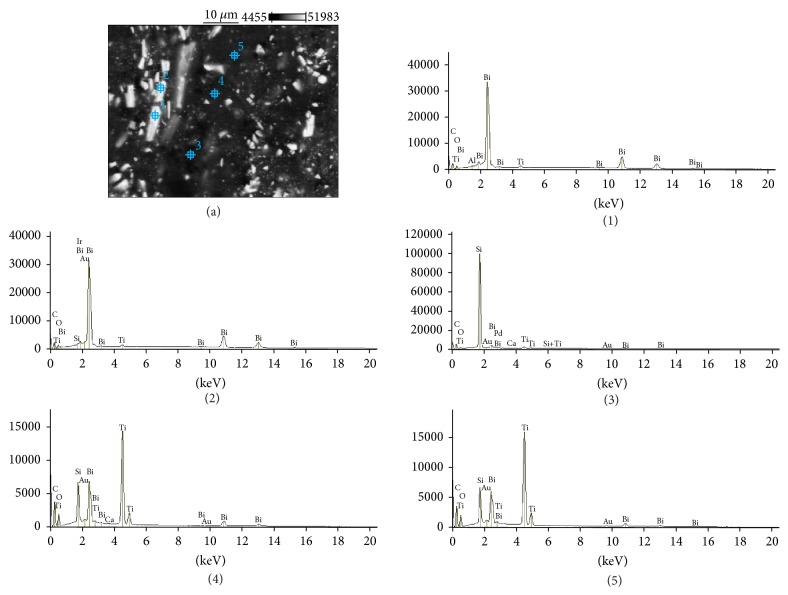
MTA Fillapex: backscatter scanning electron micrographs at 1000x magnification (a); EDS X-ray microanalysis of particles and cementation phase (1, 2, 3, 4, and 5).

**Figure 8:**
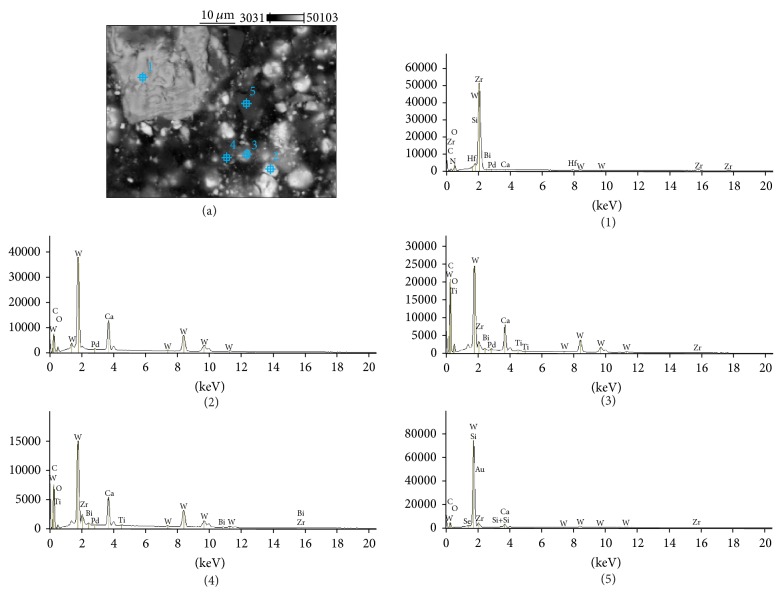
AHPlus: backscatter scanning electron micrographs at 1000x magnification (a); EDS X-ray microanalysis of particles and cementation phase (1, 2, 3, 4, and 5).

EDS analysis performed for the components of investigated areas reveals that BioRoot RCS was composed of particles rich in zirconium, hafnium, and oxygen (marked (1) and (2) in Figure 5), and the cementation phase was composed of calcium, silicon, oxygen and carbon (marked (3), (4), and (5) in Figure 5). The size of the cement particles ranged from 5 *μ*m to 30 *μ*m. Well-Root ST had a similar composition. The particles were rich in zirconium, hafnium, and oxygen (marked (1) and (2) in Figure 6). However, in contrast to BioRoot RCS, zirconium was also found in the cementation phase (marked (3), (4), and (5) in Figure 6). In this phase, peaks were also observed for oxygen, chlorine, silicon, aluminum, calcium, magnesium, and titanium. MTA Fillapex was composed of elongated particles approximately 10–15 *μ*m long that exhibited peaks for bismuth, oxygen, carbon, and titanium (marked (1) and (2) in Figure 7) and roundish particles approximately 2-3 *μ*m that exhibited peaks for titanium, bismuth, carbon, silicon, and oxygen (marked (4) and (5) in Figure 7). The cementation phase was rich in silicon, carbon, oxygen, titanium, and bismuth (marked (3) in Figure 7). AHPlus was composed of larger particles (marked (1) in Figure 8) rich in zirconium, hafnium, tungsten, carbon and oxygen, and smaller particles (marked (2), (3), and (4) in Figure 8) rich in tungsten, carbon, and calcium. Both particles were interspersed in the cementation phase composed of silicon and carbon, zirconium, and tungsten (marked (5) in Figure 8).

## 4. Discussion 

In the current study, the chemical compositions of two new calcium silicate-containing root canal sealers, BioRoot RCS and Well-Root ST, were assessed and compared to the composition of extensively studied materials: calcium silicate-containing root canal sealer MTA Fillapex and epoxy resin-based sealer AHPlus.

EDS revealed that BioRoot RCS is mostly composed of calcium, zirconium, oxygen, carbon, silicon, and chlorine. No heavy metals or other toxic elements were found in this endodontic sealer. The elements observed in the present study were biocompatible with those given by manufacturer of BioRoot RCS and determined by Camilleri in an experimental tricalcium silicate-based endodontic sealer by Septodont [32]. However, the microanalysis revealed that Well-Root ST contained aluminum and titanium in addition to calcium, zirconium, oxygen, carbon, and silicon.

In both new silicate-based root canal sealers, the particles interspersed in the cementation phase were composed of zirconium, hafnium, and oxygen, making up the radiopacifiying material. Although zirconium oxide provides a lower contrast than other radiopacifiers, such as bismuth oxide, it seems to be more inert [33].

EDS analysis of MTA Fillapex revealed that the outer surface is rich in carbon, calcium, oxygen, silicon, titanium, and bismuth, whereas AHPlus is composed of carbon, oxygen, calcium, zirconium, and tungsten. These results are in accordance with previous reports [34, 35]. However, Gandolfi and Prati observed that MTA Fillapex also contains aluminum and sulfur, and AHPlus also contains aluminum and iron [36]. The differences between cited studies may be explained by variations in the experimental conditions.

When EDS was used to confirm the chemical composition of particles interspersed in the cementation phase, MTA Fillapex exhibited peaks for bismuth, titanium, and oxide, and AHPlus for zirconium, tungsten, and oxide. These elements (bismuth oxide and zirconium oxide) are added to improve the radiopacity of endodontic sealers. According to many researchers, bismuth is associated with the discoloration of bioceramic materials and tooth tissue [37–39]. However, Ioannidis et al. compared in vitro MTA Fillapex with Roth-811 cement and found that the application of MTA-based root canal sealer results in minimal color alterations of tooth tissues (not clinically perceptible discoloration), whereas zinc oxide-eugenol cement induces severe discoloration [40].

SEM and EDS are standard methods and have been utilized previously to assess the chemical composition of root canal sealers and other endodontic materials [14, 30, 32, 33]. These methods are relatively simple and not time-consuming. However, EDS microanalysis has some limitations, including the detection of light elements and an X-ray detection limit of ~0.1% depending on the element. Therefore, some authors suggest a qualitative and quantitative overview by SEM-EDS followed by more precise ICP-OES [41].

## 5. Conclusion

Among the materials evaluated in this study, BioRoot RSC represents the highest degree of purity. The clinical implication of heavy metals contained in Well-Root, MTA Fillapex, and AHPlus needs to be investigated.

## Figures and Tables

**Table 1:** The percentage (weight%) of elements in the tested root canal sealers.

Element	Root canal sealer			
	BioRoot	Well-Root ST	MTA Fillapex	AHPlus
C	5.6–6.2	5.4–6.0	20.0–21.9	31.4–34.5
O	35.1–37.1	37.9–39.3	21.3–22.8	19.2–21.2
Si	8.4–9.4	4.6–5.4	4.5–6.9	—
Cl	1.1–1.3	0.4–0.6	—	—
Ca	25.0–26.6	21.0–22.1	0.1–0.2	4.5–4.9
Zr	20.3–22.6	22.2–27.4	—	15.1–18.6
Na	—	0.3–0.4	—	—
Mg	—	0.5–0.6	—	—
Al	—	1.9–2.5	—	—
Ti	—	1.0–2.1	11.5–15.6	0.1–0.2
Bi	—	—	34.4–38.9	0.3–0.4
W	—	—	—	22.5–24.4
